# Utilizing a low-cost desktop 3D printer to develop a “one-stop 3D printing lab” for oral and maxillofacial surgery and dentistry fields

**DOI:** 10.1186/s41205-018-0028-5

**Published:** 2018-08-13

**Authors:** Takashi Kamio, Kamichika Hayashi, Takeshi Onda, Takashi Takaki, Takahiko Shibahara, Takashi Yakushiji, Takeo Shibui, Hiroshi Kato

**Affiliations:** 1 Department of Oral and Maxillofacial Surgery, Tokyo Dental College, 1-2-2 Masago, Mihama-ku, Chiba 261-8502 Japan; 2 Oral and Maxillofacial Surgery, National Hospital Organization Takasaki General Medical Center, 32 Takamatsu, Takasaki, Gunma 371-0829 Japan; 3 Department of Oral Medicine, Oral and Maxillofacial Surgery, Tokyo Dental College Ichikawa General Hospital, 5-11-13 Sugano, Ichikawa, Chiba 271-8513 Japan; 4 Department of Endodontics, Tokyo Dental College, 1-2-2 Masago, Mihama-ku, Chiba 261-8502 Japan

**Keywords:** 3D printing, FDM 3D printer, Oral and maxillofacial surgery, Patient-specific, Accuracy, Education, Training

## Abstract

**Background:**

In the oral and maxillofacial surgery and dentistry fields, the use of three-dimensional (3D) patient-specific organ models is increasing, which has increased the cost of obtaining them. We developed an environment in our facility in which we can design, fabricate, and use 3D models called the “One-stop 3D printing lab”. The lab made it possible to quickly and inexpensively produce the 3D models that are indispensable for oral and maxillofacial surgery. We report our 3D model fabrication environment after determining the dimensional accuracy of the models with different laminating pitches (; layer thickness) after fabricating over 300 3D models. Considerations were made for further reducing modeling cost and model print time. MDCT imaging was performed using a dry human mandible, and 3D CAD data were generated from the DICOM image data. 3D models were fabricated with a fused deposition modeling (FDM) 3D printer MF-2000 (MUTOH) with a laminating pitch of 0.2 mm, 0.3 mm, 0.4 mm, or 0.5 mm. Each 3D model was then subjected to reverse scanning to evaluate the modeling conditions and deformation during modeling. For the 3D image processing system, Volume Extractor 3.0 (i-Plants Systems) and POLYGONALmeister V2 (UEL) were used. For the comparative evaluation of CAD data, spGauge 2014.1 (Armonicos) was used.

**Results:**

As the laminating pitch increased, the weight of the 3D model, model print time, and material cost decreased, and no significant reduction in geometric accuracy was observed.

**Conclusions:**

The amount of modeling material used and preparation cost were reduced by increasing the laminating pitch. The “One-stop 3D printing lab” made it possible to produce 3D models daily. The use of 3D models in the oral and maxillofacial surgery and dentistry fields will likely increase, and we expect that low-cost FDM 3D printers that can produce low-cost 3D models will play a significant role.

## Background

Three-dimensional (3D) patient-specific organ models made with 3D printing technology are utilized in various fields [1–4]. In the oral and maxillofacial surgery and dentistry fields, 3D models of hard tissues such as teeth and bones are being utilized for medical education training, explanation to the patient, operation planning, and simulated surgery using real surgical instruments [5–8]. The increased use of 3D models has directly led to an increase in the cost of obtaining them. Reducing the cost of obtaining 3D models is now one of the major concerns. By generalizing the hardware and software surrounding 3D printing technology [9, 10], a desktop fused deposition modeling (FDM) 3D printer which is extremely inexpensive compared with industrial 3D printers, we created an environment for enabling design, fabrication, and the use of patient-specific 3D models in our facility entitled the “One-stop 3D printing lab”. 3D models were produced quickly and the cost burden was greatly reduced. The laminating pitch (layer thickness) and fill density (infill density) control the amount of modeling material used. While it is expected that an increase in laminating pitch will lead to a reduction in the modeling cost, there is concern that the precision will be lowered.

In this study, we evaluated the dimensional accuracy of 3D models fabricated with different laminating pitches, aiming for a further reduction of modeling costs. In addition, based on our experience of fabricating over 300 3D models, we report on the “One-stop 3D printing lab” and investigate current problems and future prospects for further utilization of low-cost FDM 3D printers.

## Methods

**Fig. 1:**
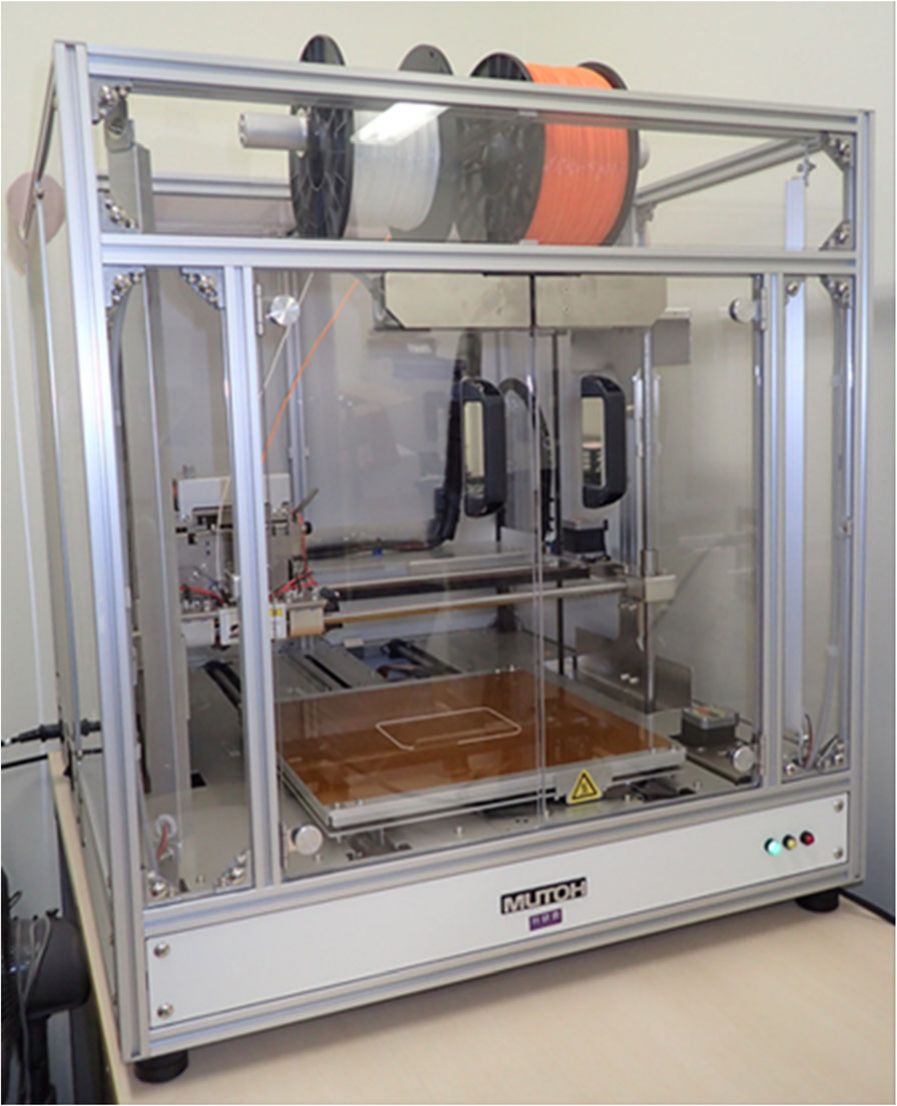
The FDM 3D printer, Value3D MagiX MF-2000

**Fig. 2:**
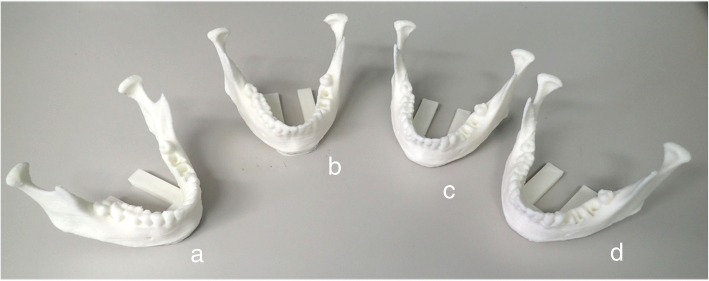
3D models with laminating pitches of 0.2 mm (**a**), 0.3 mm (**b**), 0.4 mm (**c**), and 0.5 mm (**d**)

**Fig. 3:**
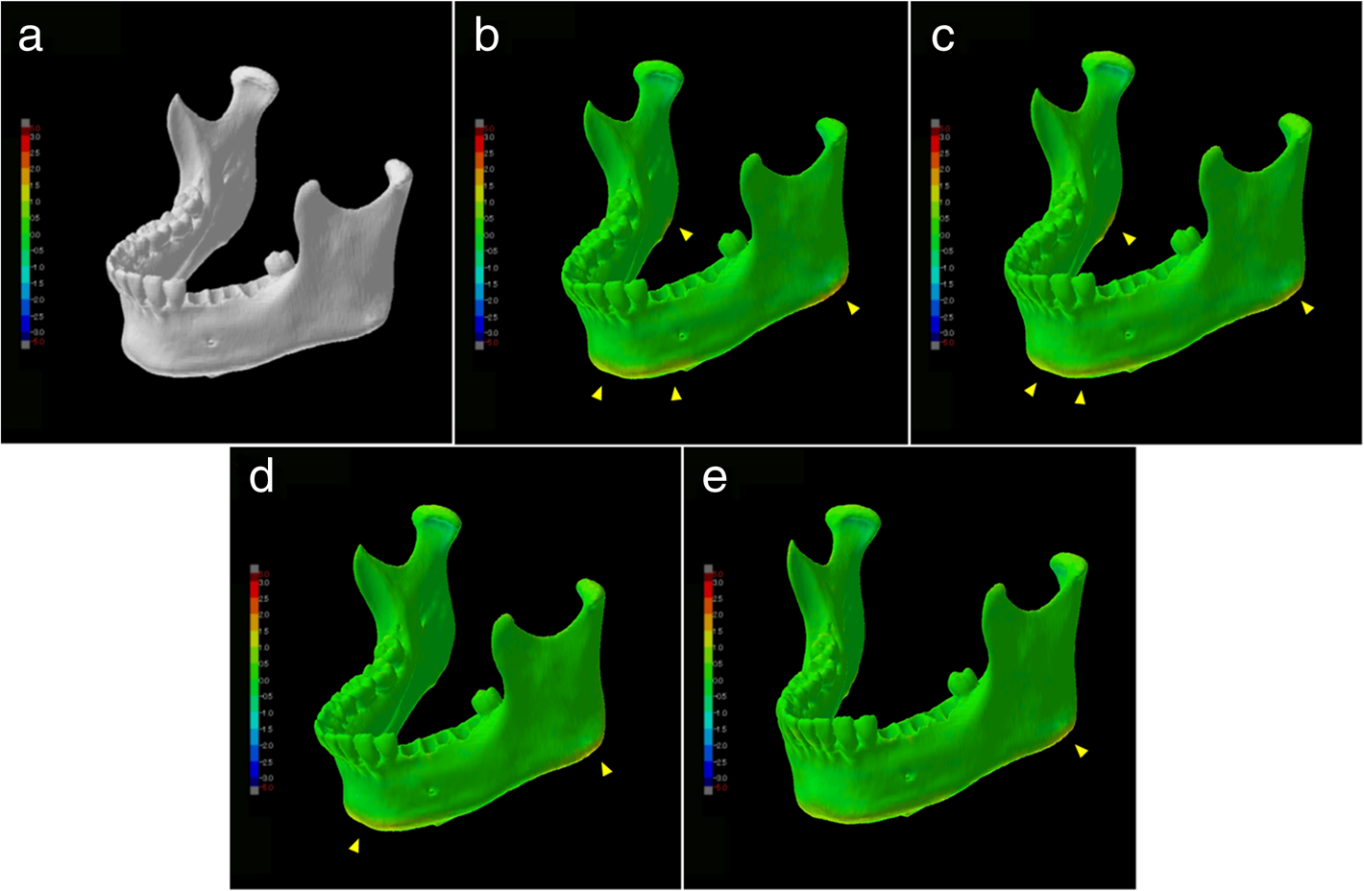
Visualization of shape error (signed differences) for each 3D CAD model. Warm color shows expansion rather than reference 3D CAD data, cold color shows shrinkage. **a** Reference 3D CAD data. **b**–**e** Slight changes in dimension were considered to be due to its own weight (arrowheads)

The shape error between the CAD model and the printed 3D object was measured to understand the printing characteristics. MDCT scanning was performed on the dry human mandibular bone, and then 3D CAD data in the STL format file (composed of about 100,000-point clouds) were created from the DICOM image data. Medical image processing software was used to create CAD data, and a desktop FDM 3D printer was used to fabricate the 3D model (Fig. 1). 3D models with laminating pitches of 0.2 mm, 0.3 mm, 0.4 mm, or 0.5 mm were created from the original 3D mandibular bone CAD data (Figs. 2a–d and 3a). MDCT reverse scanning of each fabricated 3D model was performed under the same conditions. The shape error of each 3D model against the 3D CAD model was calculated and the dimensional accuracy was evaluated.

### MDCT scanner and scanning parameters

The dry human mandibular bone was scanned and all fabricated 3D models were reverse scanned with a 64-slice MDCT (Somatom Definition AS64, Siemens, Erlangen, Germany) with the following scanning with same parameters: 120 kV tube voltage, 110 mAs, 0.6 mm slice thickness, and 128 mm FOV.

### Software and 3D printer used and evaluation procedure

The software and 3D printer used to fabricate the 3D models of the mandibular bone and the software used to evaluate the accuracy were as follows:

1. Creation of 3D CAD data from DICOM image data. A region of interest was established and binarization of images was performed with a medical imaging application (Volume Extractor 3.0, i-Plants Systems, Iwate, Japan) [11] and an STL format data editing software (POLYGONALmeister V2, UEL Corp. Tokyo, Japan) [12] was used for data volume reduction without shape change.
2. 3D printing. 3D models were fabricated using an FDM 3D printer (Value3D MagiX MF-2000, MUTOH Industries Ltd., Tokyo, Japan) based on the 3D CAD data. All models were fabricated with PLA (polylactic acid).
3. Accuracy evaluation. To evaluate the differential image processing between the 3D CAD data and the printed model and acquire a shape error (difference value), a 3D evaluation software (SpGauge 2014.1, Armonicos Co., Ltd., Shizuoka, Japan) was used.

For all comparisons, the 3D evaluation software rendered positive and negative discrepancies, which are viewed by means of a color-mapping. The color-mapping of the part comparisons were visually inspected to ascertain the specific regions of shape error (Fig. 3a–e). Evaluation of shape error (signed and unsigned differences) was carried out according to the method of Treesh et al. [13]. Each 3D CAD data was compared with the reference 3D CAD data using a best-fit registration protocol with the 3D evaluation software. Each shape error (signed and unsigned differences) included median, interquartile range, and minimum and maximum values and were recorded in millimeters.

### Statistical analysis

For evaluation of shape error between means, the data were first checked as to equal variance using the Bartlett test. If the equal variance was found, a one-way analysis of variance was used. If no equal variance was found, the Kruskal–Wallis test was employed. Comparisons within each data and among data were carried out in this way. A value of *P* < 0.05 was regarded as statistically significant. An open-source statistical analysis program, “R Ver3.5.0” was used for the statistical analysis in this study [14].

## Results

The results are shown in Table 1 and graphically in Figs. 3b, to e, 4a and b. The mean absolute shape errors of the laminating pitches of 0.2 mm, 0.3 mm, 0.4 mm, and 0.5 mm were 0.36 mm, 0.36 mm, 0.35 mm, and 0.35 mm, respectively. In the visualization of the shape error of each 3D CAD model, it is recognized that slight changes in the dimension occurred because of the own weight of the model. In particular, the tendency was found in the region of the lower edge and mandibular angles. As the laminating pitch increased, no significant reduction in geometric accuracy was observed.

**Table 1:** Outline of each fabricated 3D model and shape error evaluation with reference 3D CAD data

Laminating pitch	0.2 mm	0.3 mm	0.4 mm	0.5 mm
Model print time	4 h37 m	3 h13 m	2 h33 m	2 h17 m
3D model weight	51 g	50 g	49 g	48 g
Comparison with 3D CAD data				
Mean absolute shape error (mm)	0.36	0.36	0.35	0.35
Minimum shape error (mm)	−3.83	−3.83	− 3.78	−3.93
Maximum shape error (mm)	3.47	2.99	3.94	4.07
Standard deviation	0.53	0.53	0.56	0.58

**Fig. 4:**
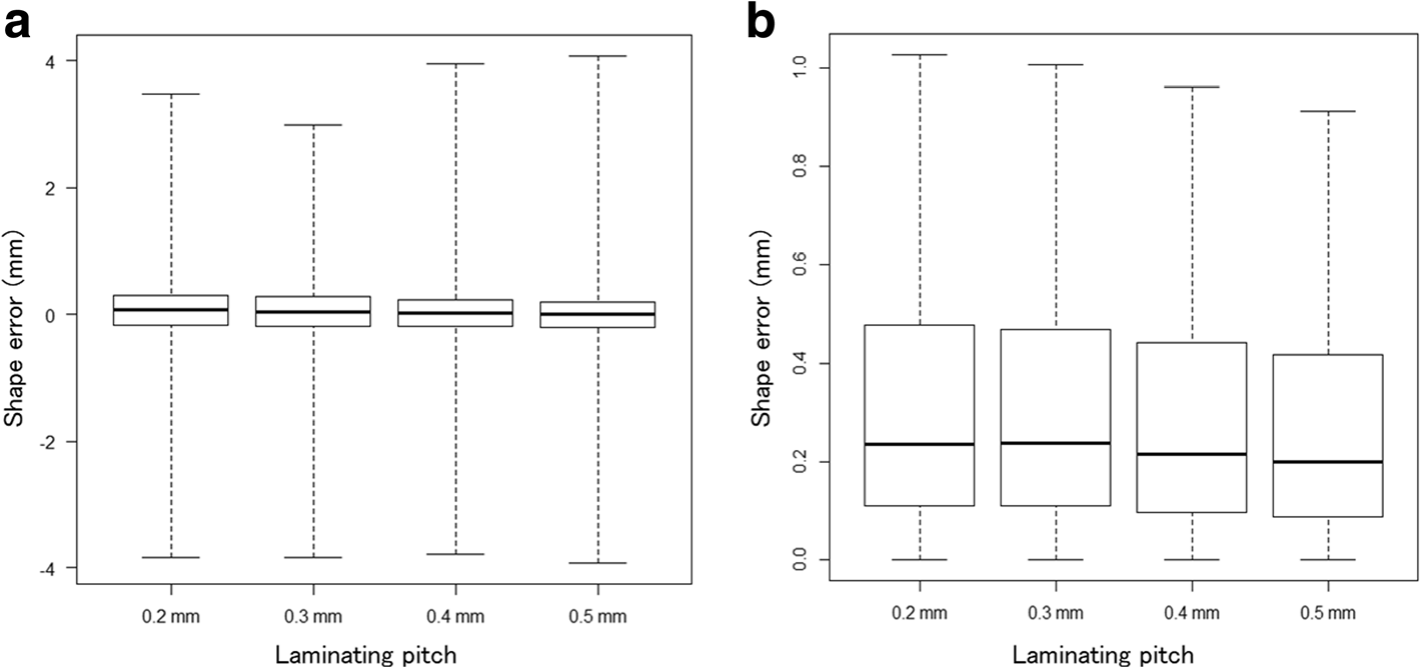
**a** Signed shape error of each 3D CAD model. The solid black line represents median value. Top of the box (upper hinge) represents 75th percentile, and bottom of the box (lower hinge) represents 25th percentile. Whiskers represent maximum and minimum values. **b** Absolute unsigned shape error of each 3D CAD model. The solid black line represents median value. Top of the box (upper hinge) represents 75thpercentile, and bottom of the box (lower hinge) represents 25th percentile. Whiskers represent maximum and minimum values

## Discussion

Despite the expense, many facilities outsource their 3D modeling to external companies because of the work and time required for their creation. If inexpensively fabricating medical 3D models were to become possible, more needs could likely be met internally. The costs of the desktop 3D printer and the modeling materials are lower than those of professional 3D printers for industrial use. To promote the spread of 3D printers in the oral and maxillofacial surgery and dentistry fields, it is essential to accumulate knowledge about the modeling characteristics of 3D printers.

### Experience using FDM 3D printers

**Fig. 5:**
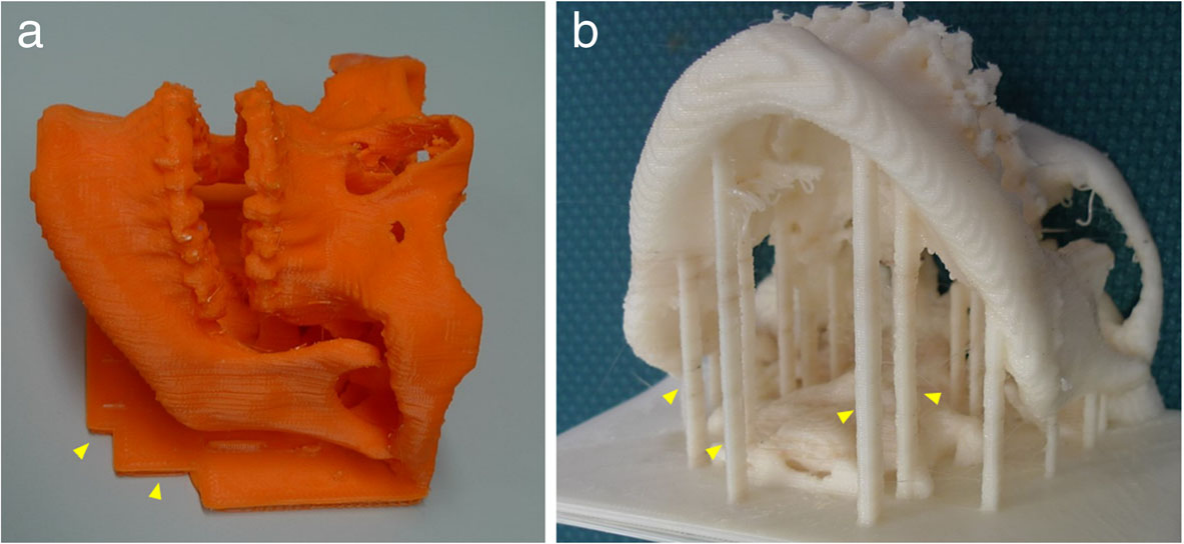
Structures fabricated as support materials (*arrowheads*). **a** To increase the contact area with the heating table of 3D printer, a plate-like support was installed. **b** To prevent deformation due to its own weight, a columnar support was installed

The results of this study show the increase in the lamination pitch means that the amount of filament to be used is reduced, resulting in shortening of model print time and reduction of cost. In recent years, 3D printers ranging from high-end printers for industrial use to low-end printers for personal use have appeared on the market. The biggest advantage of using the FDM 3D printer is that the purchase price of the equipment is low, maintenance costs are minimal, commonly used material is easy to obtain and is relatively inexpensive. Therefore, it is possible to minimize the cost of obtaining 3D models. Many of our fabricated 300 or more 3D models are PLA models with a laminating pitch of 0.3 mm and a fill density of 50%. We think that this setting has a good balance with the anatomical reproducibility we want, cost, and model print time. Models fabricated with PLA have high affinity with dental instruments and good technical workability, create a cutting feeling similar to actual bone, and can be easily used to perform the surgical simulation. In addition, it is easy to fabricate a plurality of models according to the purpose because of its low modeling cost. The disadvantages are the extrusion head of the printer must continue moving, or material bumps up and depending on the form of the 3D models, we often experience difficulties in laminating. As can be seen from this result, when distortion is expected when fabricating 3D models, in consideration of modeling direction and installation of support structures, in order to reduce deformation during the 3D CAD design stage (Fig. 5a and b).

### “One-stop 3D printing lab” for oral and maxillofacial surgery and dentistry fields

**Fig. 6:**
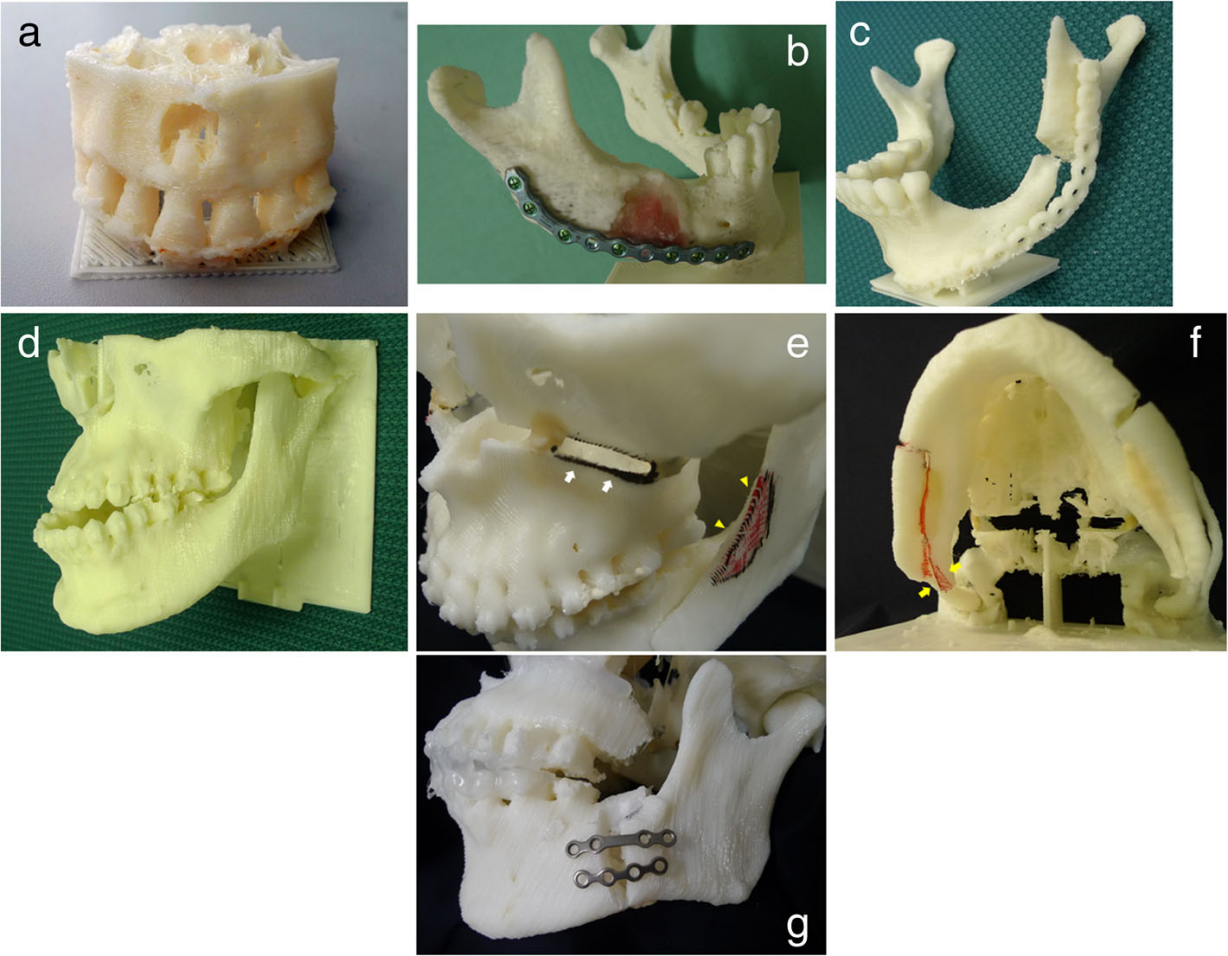
Fabricated PLA 3D models used clinically with a laminating pitch of 0.3 mm and a fill density of 50%. **a** Precise reproduction of cystic lesions and tooth roots in the maxilla. **b** Used in pre-vending of reconstruction plate. **c** Used for preoperative evaluation of secondary reconstruction of the mandible. **d** Patient-specific 3D jaw bone model of a patient with a jaw deformity. **e** Model embodies the amount of maxillary movement and direction in Le Fort I osteotomy *(white-arrow)* and bone trimming of mandibular ramus (*arrowhead*). **f** Confirmation of interferences between mandibular proximal and distal segments for the mandibular setback in sagittal split ramus osteotomy (*arrow*). **g** Fixation plates in mandibular advancement

The “One-stop 3D printing lab” in imitation of the term “One stop shop” that is a business or office where multiple services are offered, is an environment that can complete everything from design to fabricating in our facility. One of the merits of the one-stop fabrication lab is that it is possible to fabricate the model while communicating with the surgeon to determine which parts are critical in the 3D model. Figure 6a–g show our 3D modeling cases. Although the desktop/personal FDM 3D printer is often classified as low-end, it may be able to meet more expectations and allow for new developments in the oral and maxillofacial surgery and dentistry fields. However, even if the desktop/personal FDM 3D printer is introduced, it cannot be used immediately. To reproduce the different disease states in individual patients to be modeled, it is necessary to first understand the physical properties of the model, understand modality, master the software operation, and understand the dissections and readings. When we first began, 3D model creation was not quick and easy, it took significant time and effort to reduce the labor required and the number of processes in the flow from 3D CAD data construction to model output. Accumulation of know-how was a necessary part of the process.

To fabricate highly accurate 3D models, it is necessary to understand the modeling characteristics of the 3D printer used. There are many control parameters that need to be set in the 3D printer software. A trial and error process was required until a stable print protocol could be constructed. The FDM 3D printer melts materials by heat and laminates layers from the bottom. Therefore, the object may tilt under its own weight depending on the 3D shape being fabricated, and it may fall off the forming table in the middle of modeling. This was rectified by adjusting the parameters of the 3D printer control software and developing creative structural solutions during the 3D CAD data creation.

### Future prospects

Surgical simulations using a 3D model have been performed [15–18]. Mavili et al. posited that the limitations of this technology are manufacturing time and cost [16], but the desktop FDM 3D printer can solve these problems [18]. In addition to major surgery, such as bone dissection and orthognathic surgery, 3D models are also useful for treatment planning and simulation of minor surgery such as surgical endodontic treatment [19]. It has been reported that utilization of a low-end 3D printer advances the field of dental treatment, and endodontic management in particular [18–21]. Dramatic evolution of 3D printing technology is expected to further aid the dentistry field. To utilize 3D printers in the clinical practice of oral and maxillofacial surgery and dentistry, it is necessary to consider the stomatognathic field, a subspecialty of the oral and maxillofacial field. The target organs in oral and maxillofacial surgery are the teeth and jawbones, which are relatively small compared with other organs. Therefore, higher spatial resolution modalities will be necessary to obtain detailed information. In addition, the response of metal artifacts to CT imaging, which are often encountered in daily diagnostic imaging, is also a serious problem. It is often difficult to obtain information on teeth and alveolar crest bones because of metal artifacts and/or beam hardening, which may lead to an increase in the number of image processing steps or a decrease in the accuracy of the modeled objects. The CBCT which has become popular in recent years may be useful from the viewpoint of higher spatial resolution and X-ray exposure [22]. If it becomes possible to obtain high-resolution data, it will be expected to be able to fabricate more detailed 3D models. However, significant metal artifacts and beam hardening are also recognized in CBCT images. Sometimes it is more than MDCT, we experienced difficulty in 3D modeling. Unfortunately, there are as yet no measures for preventing them. For certain dental applications, a method using a hybrid model that the dental cast can be scanned and then aligned with the CT scan has also been developed [23]. However, the current situation is difficult to operate, it is difficult to say easily. Furthermore, utilizing 3D optical devices without metal artifacts is considered to be a potential solution to this challenge [13].

## Conclusions

The results obtained using the FDM 3D printer suggested that adjusting the laminating pitch may lead to further reduction of model print time and cost. It was possible to quickly print a 3D model while greatly reducing the cost burden using the low-cost desktop 3D printer in the “One-stop 3D printing lab.”

## Abbreviations

3D: Three-dimensional
CAD: Computer-aided design
CBCT: Limited cone-beam CT
CT: Computed tomography
DICOM: Digital Imaging and COmmunications in Medicine (file format)
FDM: Fused deposition modeling
FOV: Field of view
MDCT: Multidetector-row computed tomography
PLA: Polylactic acid
STL: Stereolithography (file format)
